# Foraging Burrow Site Selection and Diet of Chinese Pangolins, Chandragiri Municipality, Nepal

**DOI:** 10.3390/ani12192518

**Published:** 2022-09-21

**Authors:** Sharmila Tamang, Hari Prasad Sharma, Jerrold L. Belant

**Affiliations:** 1 Central Department of Zoology, Institute of Science and Technology, Tribhuvan University, Kirtipur, Kathmandu 44618, Nepal; 2 Nepal Zoological Society, Kirtipur, Kathmandu 44618, Nepal; 3 Department of Fisheries and Wildlife, Michigan State University, East Lansing, MI 48824, USA

**Keywords:** Chinese pangolin, prey species, foraging habitat, myrmecophagy

## Abstract

**Simple Summary:**

As one of only eight species of pangolin, the Chinese pangolin (*Manis pentadactyla*) of Nepal inhabits forests, agricultural lands, and grasslands. Its population is declining due to hunting and habitat loss, and it is listed as critically endangered. Accurate information on its habitat and diet can aid in the development of site-specific management plans. Habitat characteristics such as forest canopy cover, slopes, and distance to agricultural lands and the nearest ant nests are important factors influencing the occurrence of the Chinese pangolin. Fecal analysis revealed that the ant species *Aphaenogaster symthiesii*, *Camponotus* sp., *Monomorium* sp., and *Pheidole* sp. were the dominant prey in the Chinese pangolin’s diet. This study provides baseline information to aid Chinese pangolin conservation in Nepal.

**Abstract:**

The Chinese pangolin (*Manis pentadactyla*) is a myrmecophagous, nocturnal mammal species that occurs in forests, agricultural lands, and grasslands. It is critically endangered due to illegal hunting and habitat loss. Characterizing the Chinese pangolin’s habitat and diet could improve our knowledge of the conditions necessary for species persistence; however, limited information is available. We investigated the habitat and diet of Chinese pangolins in the Chandragiri Municipality, Kathmandu, Nepal from November 2021–March 2022. We identified foraging burrows within plots established along 20 transects, collected scats opportunistically at these burrows, and used a generalized linear model to assess the site-level habitat characteristics related to burrow occurrence. We recorded 88 foraging burrows which occurred in forests with 50–75% canopy closure at 1500–1700 m elevation with 20–40° slopes. The probability of detecting a Chinese pangolin foraging burrow was greater with the increasing slope gradient and decreased with increasing distance to agricultural lands and ant nests or termite mounds. The analysis of 10 scats revealed that *Aphaenogaster* *symthiesii*, *Camponotus* sp., *Monomorium* sp., and *Pheidole* sp. were the dominant ant prey species; no termites were detected. Baseline data from this study could be used for ex-situ conservation and the captive breeding of Chinese pangolins as well as aiding site-specific management plans in Nepal.

## 1. Introduction

The foraging site selection and the diet of species provide insights into their ecology and habitat use [1]. The knowledge of foraging ecology and diet is essential for developing conservation action and management plans for species’ long-term persistence [2,3] and their impacts on the prey species [4]. This knowledge is particularly important for species that are endangered or are of conservation concern.

The Chinese pangolin (*Manis pentadactyla*, Linnaeus 1758) is one of the four Asian pangolin species and occurs in Nepal, India, China, Taiwan, Bangladesh, Bhutan, Myanmar, Lao PDR, Thailand, and Vietnam [5]. The Chinese pangolin occurs in primary and secondary tropical forests [6], grasslands, agricultural areas, and some degraded habitats [7,8,9,10,11]. Chinese pangolins tend to inhabit broad-leaved forests because of the greater abundance of termites [12]. However, their occurrence can also be influenced by elevation, slope gradient, canopy cover, and the distance to water and human activity [9,13,14,15]. Chinese pangolins are nocturnal and have adapted for digging burrows which are used for hunting prey, shelter, and avoiding predators [12,16].

The Chinese pangolin is considered vulnerable to human threats due to its low reproductive rate, poor self-defensive mechanisms, and narrow habitat requirements [12]. It is classified as Critically Endangered by the International Union for Conservation of Nature (IUCN) Red List of Threatened Species [5]. The Chinese pangolin is listed under Appendix I of the Convention on International Trade in Endangered Species of Wild Fauna and Flora [17] and protected in Nepal under the National Parks and Wildlife Conservation (NPWC) Act 1973 [18]. Human threats to the Chinese pangolin include illegal hunting for its flesh and scales [5] as well as habitat alteration and degradation [7,11,19]. Pangolins are among the few myrmecophagous mammal species [20], with specialized anatomical and morphological adaptations for foraging primarily on termites and ants [21,22,23,24]. They opt for specific ant and termite species rather than foraging on the most abundant species [23,25,26]. The Chinese pangolin selects ants over termites as prey, feeding on over 70 species of ant and 4 termite species [27]. Dominant ant prey species include *Pheidologeton yanoi*, *Pheidole nodus, Polyrachis fervens*, *Crematogaster schimmeri*, *Camponotus monju*, and *Pseudolasius binghami*, and the dominant termite prey species is *Odontotermes formosanus*. Chinese pangolins also appear to help control the invasive ant *Anoplolepis gracilipes* [28]. Therefore, the conservation of the Chinese pangolin could help maintain ecosystems by regulating insect populations [5,19] and provide ecosystem services by improving soil quality and mitigating the crop damage caused by termites and ants [8,29].

Because of their nocturnality, adaptations for burrowing [30,31], and their tendency to forage 5–6 km from their resident burrows each night [32], the foraging behavior and ecology of pangolins are difficult to observe in the field. Their specialist diet also makes them difficult to maintain in captivity for observational studies [33]. Consequently, there is limited knowledge regarding the foraging ecology and diet of the Chinese pangolin. We characterized the foraging burrow site selection of the Chinese pangolin and identified prey remains from scats associated with these burrows from the Chandragiri Municipality, Nepal, to further our understanding of pangolin ecology and improve its conservation.

## 2. Materials and Methods

### 2.1. Study Area

**Figure 1:**
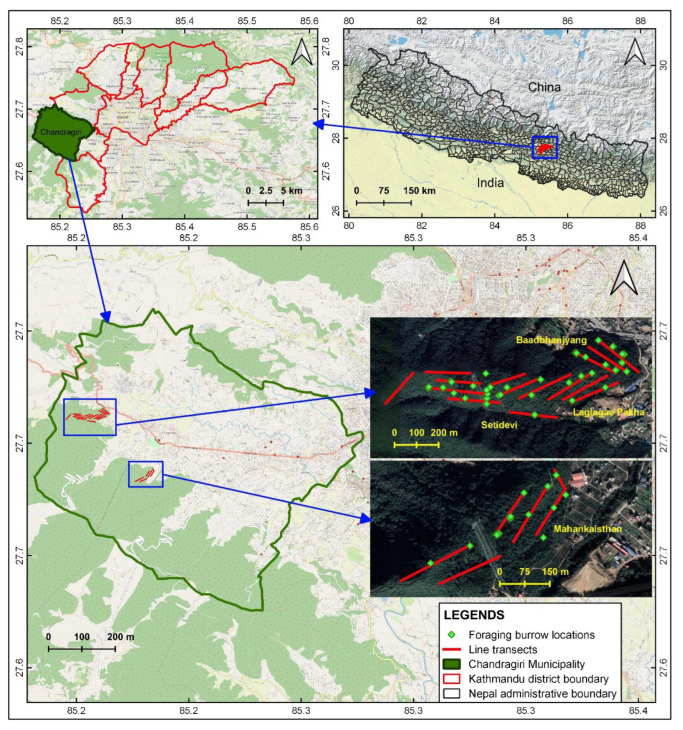
Chinese pangolin study area with established line transects and foraging burrows in Chandragiri Municipality, Nepal.

The Chandragiri Municipality (27°43′36.49″–27°32′45.03″ N, 85°16′39.51″–85°11′8.68″ E) is in southwest Kathmandu District, Bagmati Province, Nepal (Figure 1) and comprises 43.9 km^2^. It has a human population of 136,928 (3118 people/km^2^; [34]). It has predominantly hilly terrain with elevations from 1310 to 2551 m above sea level. It contains 23 community forests covering 1171 ha. The vegetation is mixed forest and includes the Nepalese alder (*Alnus nepalensis*), needlewood (*Schima wallichii*), chinkapin (*Castanopsis tribuloides*), pine (*Pinus roxburghii*), oak (*Quercus* spp.), rhododendron (*Rhododendron arboretum*), Himalayan ash (*Fraxinus floribunda*), and marking nut (*Semicarpus anacardium*). Major mammal species include the large Indian civet (*Viverra zibetha*), yellow-throated marten (*Martes flavigula*), jungle cat (*Felis chaus*), golden jackal (*Canis aureus*), Chinese pangolin (*Manis pentadactyla*), hoary-bellied squirrel (*Callosciurus pygerythrus*), leopard cat (*Prionailurus bengalensis*), leopard (*Panthera pardus*), and wild boar (*Sus scrofa*) [35].

### 2.2. Methods

We conducted our field survey from November 2021–March 2022 within the Mahankal, Setidevi, Laglagae, and Baadbhanjyang community forests in the Chandragiri Municipality. Within each forest we established five transects (20 total) about 300 m long and established five, 10 × 10-m plots on each transect at 50-m intervals (Figure 1). We established a total of 100 survey plots on the 20 transects. In each plot, we recorded the foraging burrow occurrence, collected the fecal samples observed, and measured eight habitat covariates. We counted the number of ant nests and termite mounds within each plot and measured the distance from the plot center to the nearest nest or mound. We estimated the slope gradient using a clinometer at the plot center. We estimated canopy coverage by averaging values obtained at the plot center and the four corners using a spherical densiometer. We counted the number of trees (>5-cm diameter at breast height and >1.5-m tall) in each plot. We then measured the distance from the plot center to the nearest road, water source (e.g., stream, pond), and agricultural land; distances <25 m were measured using a tape measure, with greater distances estimated using Google Earth Pro.

We collected Chinese pangolin fecal samples opportunistically from burrow openings to estimate diet composition, placing each in a plastic bag with desiccant before analyses. We identified fecal samples from the Chinese pangolin by visual observation and by detecting the presence of chitin fragments in the feces [36].

In the laboratory, we placed samples in 70% ethanol and separated items manually, and then used a stereomicroscope for identification. We identified prey to the lowest taxonomic level using keys for ants [37], termites [38], and other invertebrates, as well as a reference collection obtained during our surveys in the study area.

**Figure 2:**
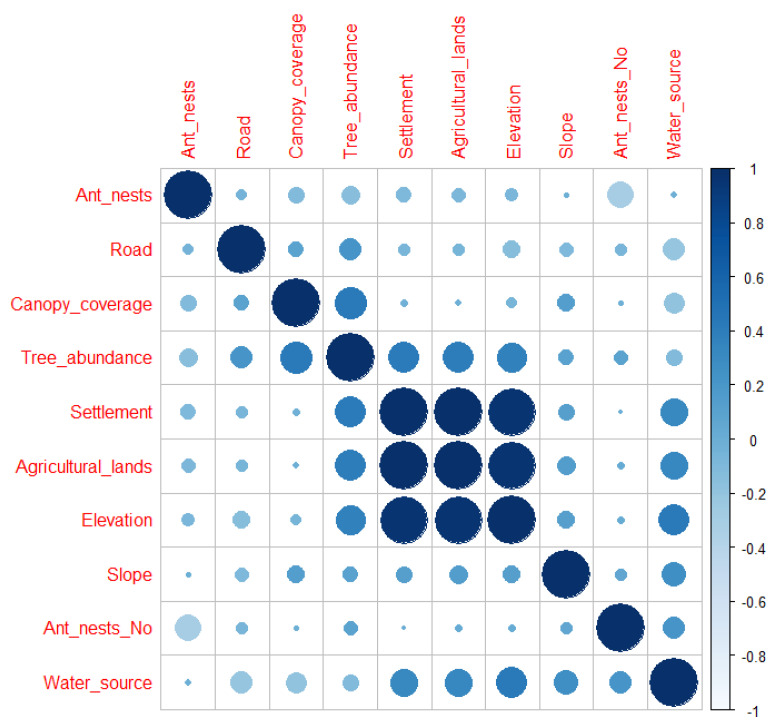
Correlation matrix between predictive variables to estimate factors influencing Chinese pangolin foraging burrow occurrence in Chandragiri Municipality, Nepal. Variables Ant_nests = distance to nearest ant nest, Road = distance to nearest road, canopy_coverage = % forest canopy coverage, Tree_abundance = number of trees, Settlement = distance to nearest settlement, Agricultural_land = Distance to nearest agricultural land, Ant_nest_no = number of ant nests and water_source = distance to nearest water source.

We used a generalized linear mixed model to identify factors affecting the occurrence of the Chinese pangolin in the Chandragiri Municipality in 2021. Factors include elevation (m), slope gradient (°), forest canopy cover (%), distance to a water source (m), roads (m), settlements (m), agricultural land (m), ant nests (m), no. of ant nests, and tree abundance. Elevation and distance to settlement were highly correlated variables (|r| > 0.7) with distance to agricultural land, and we retained the latter for analysis (Figure 2). We rescaled continuous variables from 0 to 1 before analyses. We used R program for analyses [39]. Means are reported with a +1 standard error (SE).

## 3. Results

**Figure 3:**
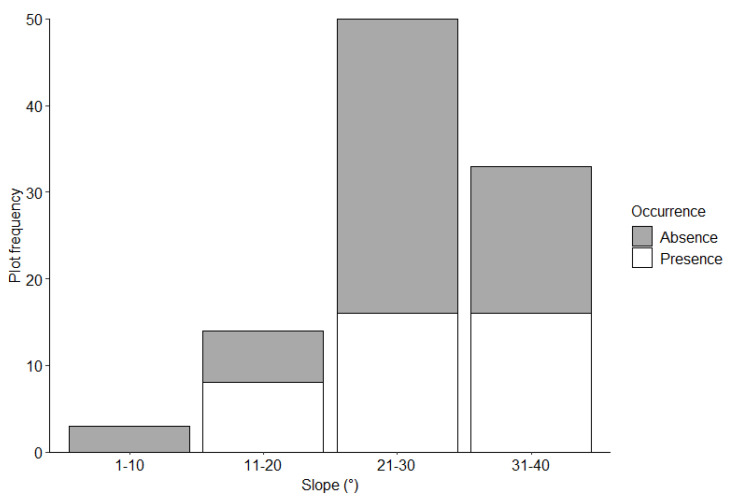
Chinese pangolin foraging burrow distribution by slope gradient in Chandragiri Municipality, Nepal.

**Figure 4:**
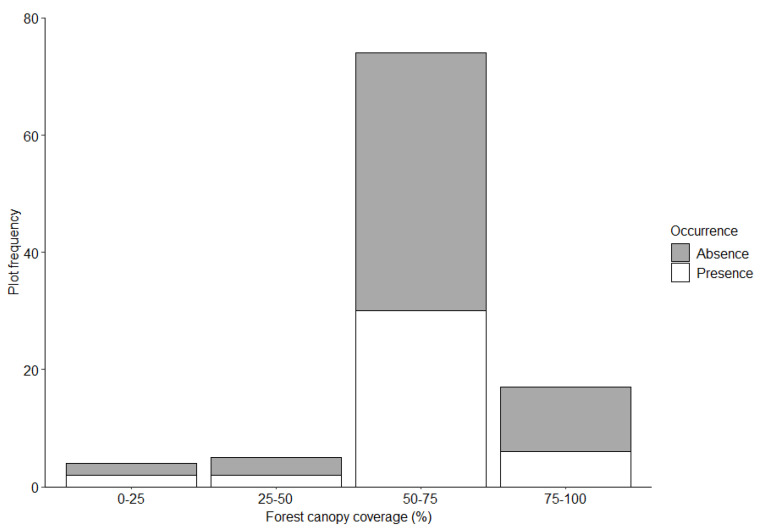
Chinese pangolin foraging burrow distribution by forest canopy coverage in Chandragiri Municipality, Nepal.

**Figure 5:**
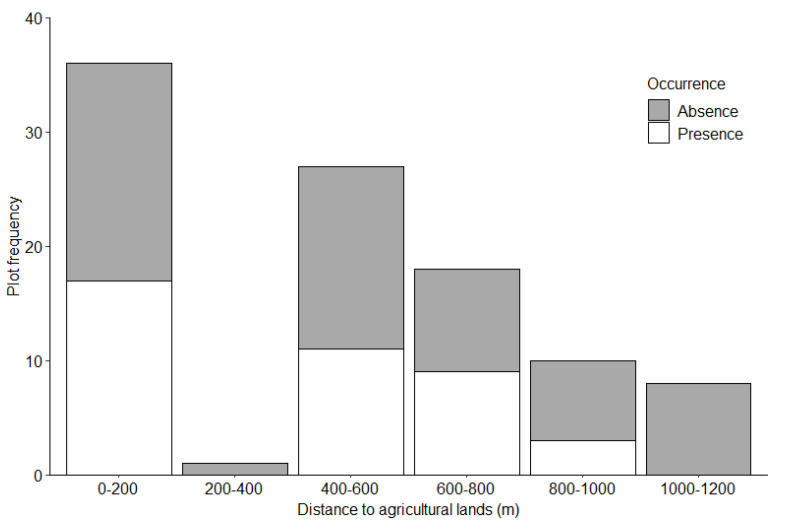
Chinese pangolin foraging burrow distribution by distance to agricultural lands in Chandragiri Municipality, Nepal.

**Figure 6:**
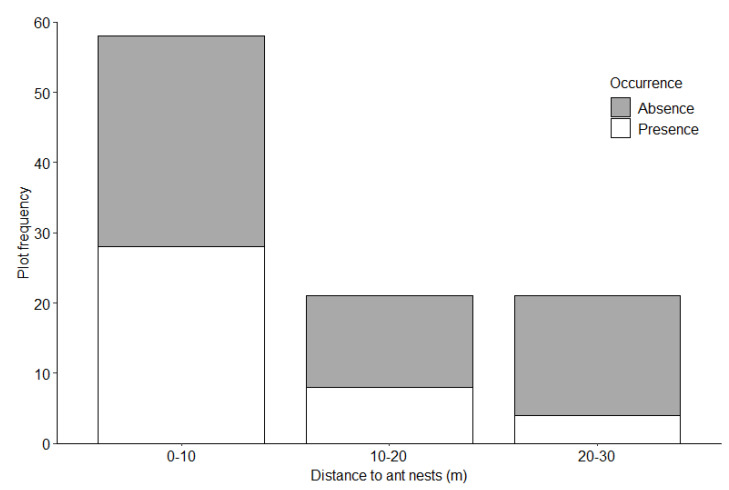
Chinese pangolin foraging burrow distribution by distance to ant nests in Chandragiri Municipality, Nepal.

We identified 88 foraging burrows in the 38 surveyed plots; 30 (34%) in the Mahankalsthan, 15 (17%) in the Setidevi, 24 (27%) in the Baadbhanjyang, and 19 (22%) in the Laglagae Pakha community forests. We located burrows 1450 to 1800 m above sea level [(average 1585 ± 11.6 m (SE)] with most burrows (81.8%, *n* = 81) within 1500–1700 m elevation (Table 1). Most burrows (46.5%, *n* = 46) were recorded on 30–40° slopes (Figure 3). Most burrows (61.4%, *n* = 54) were under 50–75% forest canopy coverage (Figure 4). The average distance to the nearest road from the center of the plot was 28.1 ± 0.9 m (range = 1 to 272.3 m), and most burrows (88.6%, *n* = 78) were <100 m from the nearest road. Most burrows (55.6%, *n* = 55) were <100 m from the nearest water source. The average distance to agricultural lands was 224 ± 28.2 m (range = 55–1025 m) with most burrows (78.8%, *n* = 78) recorded within 400 m of agricultural lands (Figure 5). The average distance to the nearest ant nest or termite mound from the center of plots was 5.4 ± 1.3 m (range = 0.6–38 m) with most burrows (72.7%, *n* = 72) within 20 m (Figure 6).

The probability of detecting a Chinese pangolin foraging burrow was greater with increasing slope and decreased with increasing distance to agricultural lands and ant nests or termite mounds (Table 2). No other variables measured were significant.

**Figure 7:**
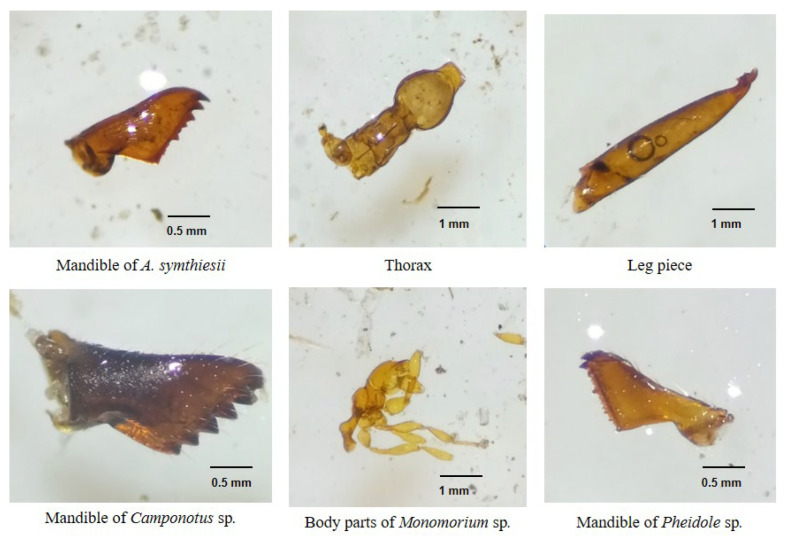
Ant body parts found in the fecal samples of the Chinese pangolin in Chandragiri Municipality, Nepal.

**Figure 8:**
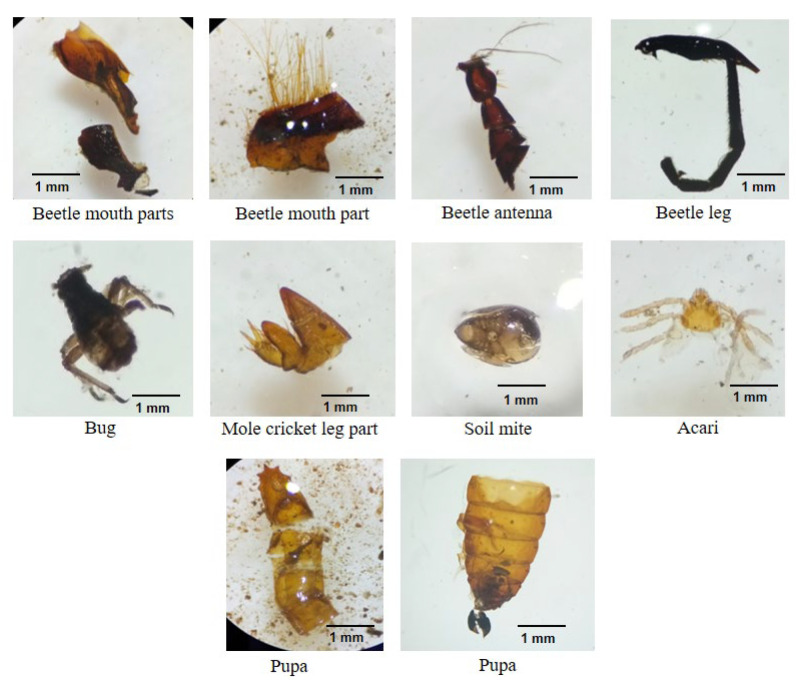
Insect body remains from fecal samples of Chinese pangolins in Chandragiri Municipality, Nepal.

**Figure 9:**
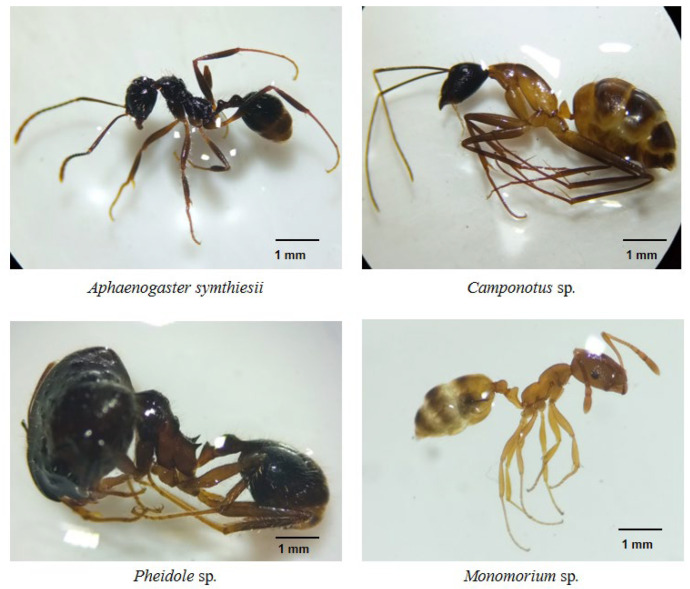
Major ant species present in the diet of Chinese pangolins in Chandragiri Municipality, Nepal.

We collected 10 Chinese pangolin fecal pellets. The prey species detected were comprised solely of invertebrates including *Aphaenogaster symthiesii*, *Camponotus* sp., *Monomorium* sp., *Pheidole* sp., beetles, soil mites, a bug, a mole cricket, and an unidentified pupa (Figure 7, Figure 8 and Figure 9).

## 4. Discussion

The occurrence of Chinese pangolin foraging burrows was influenced by slope gradient, distance to agricultural lands, and distance to ant nests. The presence of burrows on slopes of 30–40° suggests moderately steep slopes are more suitable for foraging. Steeper slopes (30–60°) could maintain stable temperatures inside burrows and ensure the availability of termites [40], whereas the absence of burrows in slopes >60° could be due to reduced food availability and accessibility [12,40,41]. Moderately steeper slopes could also ensure the stability and integrity of burrows by reducing erosion and facilitate their excavation [40].

We found that canopy cover influenced pangolin burrow presence; that most burrows occurred in forests with 50–75% canopy coverage suggests that Chinese pangolins opt for intermediate to higher levels of canopy cover, as reported previously [9]. Chinese pangolins have poor defenses against predators [42], and a greater canopy cover could reduce predation risk [43]. Chinese pangolins have a poor capacity to adapt to changing temperatures, and denser canopy cover may also buffer against fluctuations in ambient temperature [40]. Additionally, ants and termites are more abundant and diverse in areas with denser forest canopies [44,45]. Finally, forests with denser canopies have less understory vegetation which could facilitate pangolin movements [46].

The occurrence of Chinese pangolin foraging burrows was greater nearer to agricultural areas, but the distance to water and roads did not influence burrow occurrence. Agricultural lands are abundant with ants and termites due to the presence of plant debris and animal dung [47]. However, anthropogenic activities such as the collection of fuel wood and fodder, livestock grazing, and the use of pesticides can cause disturbance and decreased prey availability for pangolins [15]. Moreover, pangolin activity near agricultural or other areas of human activity could increase their risk of being hunted [48]. The lack of relationship between the distance to water and burrow occurrence could be a consequence of the prevalence of water in the study area. Pangolins use water sources frequently and ants, termites, and other insects often select moist habitats [49,50]. We suggest that the abundance of water sources in our study area alleviated the need for the spatial selection of this resource.

The greater presence of Chinese pangolin foraging burrows near ant or termite mounds is undoubtedly a consequence of their myrmecophagous diet [12,43]. Burrows appear to be excavated near food sources [HPS personal observation] which would reduce energy expenditure when foraging [51]. Chinese pangolins feed almost exclusively on ants and termites with occasional feeding on other invertebrates. The presence of only four ant species (*A. symthiesii*, *Camponotus* sp., *Monomorium* sp., and *Pheidole* sp.) in the fecal samples despite that 15 ant species were collected from the study area as references suggest that the Chinese pangolin may exhibit selection among ant species. Several reasons could explain the species consumed: (1) pangolins may feed on the most available species; (2) pangolins may opt for larger (>5 mm body length) species. Larger ants could increase foraging efficiency and provide more energy and nutrients than smaller-sized prey [26]; and (3) pangolins may prioritize easy-to-capture prey [25,28]. Ant species recovered from scats in this study nest just below the soil surface or in decomposed logs which can be readily obtained by pangolins, particularly juveniles [28].

The selection of the ant species consumed might also be due to the chemical and mechanical defenses of the prey species; pangolins avoid ant and termite species with well-developed defense systems [52]. Ant species in the subfamily Ponerinae have strong defenses against predators [28], which could explain the avoidance of Ponerinae species such as *Brachyponera* and *Ectomomyrmex* by the pangolins. Previous studies suggest that pangolins prefer ants over termites [23,25,26,27] for reasons including difficulty accessing termites from mounds [41], which may partially explain why termites were not detected in Chinese pangolin fecal samples in this study. However, the complete digestion of termites that are relatively soft-bodied compared to ants could have limited our ability to detect them [53].

## 5. Conclusions

The foraging habitat selection of Chinese pangolins was influenced by slope and apparent food availability. Chinese pangolins may exhibit prey selection, but larger studies quantifying prey use and availability are required. Our study provides baseline data on the foraging habitat use and diet that could benefit ex-situ conservation efforts as well as captive breeding programs for Chinese pangolins. Furthermore, this information can be used to aid site-specific management plans in Nepal to protect or improve habitat suitability for Chinese pangolins.

## Figures and Tables

**Table 1:** Habitat variables for sites with (*n* = 38) and without (*n* = 62) Chinese pangolin foraging burrows, Chandragiri Municipality, Nepal.

Variable	With Burrow			Without Burrow		
	Mean	SE	Range	Mean	SE	Range
Elevation (m)	1585	11.6	1461–1737	1610	15.3	1454–1886
Slope (°)	28.1	0.9	16–40	24	1.1	8–40
Forest canopy coverage (%)	66.8	2.5	1.5–80.8	65.8	1.6	12.2–82
Distance to road (m)	40.8	8.8	2–272.3	18.5	6.4	1–239.7
Distance to water (m)	93.3	14.1	5.5–376.9	73.1	16.9	1.8–536.5
Distance to settlement (m)	284.3	26.2	50–939.8	233.7	35.4	47.3–656.6
Distance to agricultural lands (m)	245	28.2	57.8–694.6	329.2	37.3	63.8–1024.8
Number of ant nests	1.6	0.2	1–8	1	0.1	1–4
Distance to ant nest (m)	5.4	1.3	0.6–38	10	1.6	0.5–48
Tree abundance	13.1	1.03	1–29	12.5	0.7	3–27

**Table 2:** Generalized linear mixed model estimates and 95% confidence limits describing the Chinese pangolin occurrence in Chandragiri Municipality, Nepal. Variables include slope gradient (°), forest canopy coverage (%), distance to water source (m), road (m), agricultural land (m), and ant nests (m), no. of ant nests, and tree abundance were included in model construction. Significant effects (*p* < 0.05) are in bold.

Variables	Estimate	Standard Error	Z-Score	*p*
(Intercept)	0.928	1.297	0.716	0.474
Slope gradient (°)	2.612	1.117	2.338	**0.019**
Forest canopy coverage (%)	−3.260	1.640	−1.988	**0.047**
Distance to road (m)	1.483	1.198	1.238	0.216
Distance to water source (m)	−0.291	1.384	−0.210	0.834
Distance to agricultural land (m)	−3.224	1.180	−2.731	**0.006**
Distance to ant nest (m)	−2.720	1.203	−2.261	**0.024**
Number of ant nests	1.099	1.869	0.588	0.557
Tree abundance	2.385	1.504	1.586	0.113

## Data Availability

The data presented in this study are available on request from the corresponding author.
